# Long-term safety of mid-urethral sling for stress urinary incontinence in women: an emulated trial using French national health data system

**DOI:** 10.1016/j.eclinm.2025.103411

**Published:** 2025-09-15

**Authors:** Cyrille Guillot-Tantay, Sylvie Guillo, Minh Hoang Thuy Diep Tran, Yann de Rycke, Agnès Dechartres, Pierre Mozer, Emmanuel Chartier-Kastler, Florence Tubach

**Affiliations:** a Hôpital Foch, Service d'urologie et de transplantation rénale, F92150, Suresnes, France; b Sorbonne Université, INSERM, Institut Pierre Louis d’Epidémiologie et de Santé Publique, Équipe PEPITES, F75013, Paris, France; c AP-HP, Hôpital Pitié Salpêtrière, Département de Santé Publique, Centre de Pharmacoépidémiologie (Cephepi), CIC-1901, F75013, Paris, France; d Sorbonne Université, Hôpital Pitié Salpêtrière, Service d'urologie, AP-HP, F75013, Paris, France; e Inserm U1179 Handicap Neuromusculaire (UVSQ): Physiopathologie, Biothérapie et Pharmacologie appliquées, Equipe: Biothérapie & Pharmacologie des Dysfonctions Urogénito-sexuelles d'origine Neurologique, France

**Keywords:** Urinary incontinence, Stress urinary incontinence, Mid urethral slings, Safety, Target trial emulation

## Abstract

**Background:**

Mid-urethral sling (MUS) is the most-indicated therapy for treating stress urinary incontinence (SUI) in women. However, serious long-term side effects have escaped post-marketing studies. We aimed to compare two different MUS types, tension-free vaginal tape (TVT) and trans-obturator tape (TOT), in terms of incidence of MUS removal or section after implantation.

**Methods:**

We emulated a hypothetical target trial using French national healthcare data (SNDS) and included women who had undergone a first MUS implantation from 2011 to 2018 (Health Data Hub registration no. T16166982020061). The primary outcome was the cumulative incidence of MUS removal or section. Secondary outcomes were the cumulative incidence of MUS removal and section, hospitalization for urinary retention and MUS erosion and infection, and any of these outcomes, as well as reoperation for SUI. We used propensity score-weighted Cox models.

**Findings:**

In all, 215,141 women underwent implantation: 170,781 (79.4%) with TOT and 44,360 (20.6%) with TVT. At 5 years, the weighted cumulative incidence of MUS removal or section was lower in the TOT than TVT group (3.25%, 95% confidence interval CI 3.16–3.34 versus 4.13%, 95% CI 3.94–4.33). We observed a time-varying effect ([0–3 months] after implantation [hazard ratio 1.65, 95% CI 1.52–1.78], [3–12 months] after implantation [1.19, 95% CI 1.06–1.32], [1–5 years] after implantation [0.98, 95% CI 0.88–1.1] and ≥5 years after implantation [1.44, 95% CI 1.13–1.85]). MUS removal and section and hospitalization for urinary retention and MUS erosion and infection were significantly more frequent in women with TVT than TOT but second MUS implantation for SUI recurrence was less frequent.

**Interpretation:**

The risk of MUS removal or section was higher after TVT than TOT, with a time-varying effect, in this hypothetical target trial.

**Funding:**

This study was funded by a grant from the French Ministry of Health.

**Research in context**

**Evidence before this study**

To evaluate the long-term safety outcomes of synthetic mid-urethral sling (MUS) in adult women with stress urinary incontinence, we performed a literature search of MEDLINE via PubMed for articles published from January 1, 1996, when the first MUS was described, to May 31st, 2025, considering only studies with at least 5 years of follow-up and the two MUS types: tension-free vaginal tape (TVT) and trans-obturator tape (TOT).

In the 45 studies analyzed, the results were heterogeneous across studies: the rate of reoperation ranged from 0% to 19% at 5 years and 0%–17% at 10 years, depending on the study and the sling implanted. Published studies did not allow us to indicate which slings had the best safety profile.

**Added value of this study**

This is the first study reporting real-life data on long-term complications of MUS from all synthetic MUS implantations, TVT or TOT, performed in France for female stress urinary incontinence from 2011 to 2018. The cumulative incidence of MUS removal or was higher in the TVT than TOT group. We also found more MUS removal and section, hospitalization for erosion, urinary retention or MUS infection but also more frequent urinary infection with TVT than TOT but less frequent use of painkiller after 1 year and less secondary MUS implantation for stress urinary incontinence recurrence.

**Implications of all the available evidence**

This emulation study showed that complications from MUS implantation may occur even more than 5 years after implantation. These results could call into question the surgical approach to MUS implantation, for a first implantation. This preference must be balanced with the type of complications women may experience, focusing on not only the reoperation rate but also painkiller use and urinary infections.

## Introduction

According to the International Continence Society, stress urinary incontinence (SUI) is defined as the complaint of involuntary loss of urine on effort or physical exertion, including during sporting activities or on sneezing or coughing.^1^ Appearing 30 years ago, mid-urethral sling (MUS) became the most indicated therapy for this functional disease by the 2010s. There are two types of MUS: tension-free vaginal tape (TVT) and trans-obturator tape (TOT), implanted according to the operator's preference. TVT was the first developed, in 1990, by Ulmsten (1996 in France). TOT started to be used in the early 2000s.^2^ Because of a greater risk of bladder injury with TVT than TOT, its implantation requires the intraoperative cystoscopy, so it is mainly implanted by urologists, whereas TOT is implanted by both urologists and gynaecologists.^3^^,^^4^

According to systematic reviews, intraoperative and short-term post-operative complications (during the first year after implantation) seem higher with TVT than TOT (possibly because of a longer learning curve).^5^ In contrast, mid- (from 1 to 4 years) and long-term (≥5 years) complications were found more common with TOT than TVT.^6^ However, for both TVT and TOT, long-term safety data are scarce and heterogeneous, and no large-scale direct comparative studies have been performed.^7^

Most complications after MUS implantation have been described within 2 years of the implantation.^8^ This observation is currently debated because serious long-term side effects have escaped post-marketing studies. For a few years, in the United States, Canada and Australia, several “class action suits” have been taken against manufacturers marketing these synthetic MUS and pelvic floor mesh, in relation to complications that are sometimes serious (MUS erosion and/or infection, urinary retention and infection, overactive bladder, chronic pelvic pain).^9^ In this context, some countries such as the United Kingdom have even removed MUS from their practice guidelines.^10^ Because of no head-to-head comparisons with long-term follow-up to date, comparing the complications of both techniques over the long term would be invaluable for informing shared decision-making, by improving knowledge of the risk–benefit balance.

Healthcare databases are of great interest to investigate safety issues in real life settings because they cover large and unselected populations, in the context of usual care covering all type of practices with long-term follow-up. The main objective of this study using French national healthcare data was to compare two different MUS types, TVT and TOT, in terms of the incidence of MUS removal or section after implantation.

## Methods

In this study, we emulated a hypothetical target trial with real-world data. First, we designed a randomized controlled trial that would have been set up to answer the causal question, and second, we emulated the trial as closely as possible by using observational data from the French national healthcare data system (SNDS).

### Target trial

The hypothetic target trial was a randomized controlled trial aiming to compare TVT and TOT in terms of long-term safety. Eligibility criteria were women scheduled for MUS implantation for SUI with no history of previous MUS implantation. Participants were randomly assigned to TVT or TOT. Participants and their treating physicians were aware of the assigned treatment strategy. Indeed, the scars were in different places depending on the approach (thigh roots for TOT, supra-pubic for the TVT). The primary outcome was the cumulative incidence of MUS removal or section (composite outcome) (i.e., surgical procedures related to the implanted MUS). Secondary outcomes were the cumulative incidence of MUS removal and section, hospitalization for urinary retention and MUS erosion and infection, and the occurrence of at least one of these five outcomes. Criteria to assess the efficacy of MUS were the cumulative incidence of reoperation for SUI, the number of pads used in 24 h and the resolution of incontinence (patient-reported outcomes). We also considered other complications (chronic pain, overactive bladder, urinary tract infection), separately in the first year after implantation and thereafter. This 1-year period after implantation corresponds to the consolidation phase that occurs after the initial stages of inflammation and healing. Patients are followed from randomization to the occurrence of the outcome of interest, death, lost to follow-up or the end of the follow-up period (with a follow-up of at least 1 year and up to 10 years). The causal contrast of interest was an intent-to-treat effect, and the treatment effect was estimated with a Cox proportional-hazards model after checking the proportional-hazards assumption.

### Data source

The SNDS is the French national health data system. It includes the national healthcare insurance schemes and hospital discharge database, linked to each patient by a unique identifier. For each insured resident, the SNDS provides data on drug and device deliveries, tests and procedures received during ambulatory care as well as information related to hospitalizations (diagnoses, procedures). Data are available on date of birth, place of residence, sex, and long-term disease (LTD) that allows 100% reimbursement for serious and costly LTDs.^11^ Since 2006, these data, covering 99% of the entire French population (∼67 million inhabitants), have been prospectively and exhaustively recorded.

Eligibility criteria, outcomes and other covariates for the study were identified by procedure codes (Classification Commune des Actes Médicaux [CCAM]) or diagnostic codes (International Classification of Diseases, 10th Revision [ICD-10]) in the hospital discharge summaries or for LTD status, or Anatomical Therapeutic Chemical (ATC) classification codes (international standardized classification of drugs). The codes used are in Supplemental 1.

### Emulation of the target trial

We used data from the SNDS database to emulate this target trial. This study was conducted according to the European Centre for Pharmacovigilance and Pharmacoepidemiology (ENCePP) methodological standards and code of conduct (https://encepp.europa.eu/index_en). The reporting follows the STROBE and RECORD-PE guidelines.

#### Ethics

This study is part of a larger research program registered on the Health Data Hub website (no. T16166982020061), approved by the committee for research, studies, and evaluations in the field of health (Comité d’Expertise pour les Recherches, les Etudes et les Evaluations dans le domaine de la Santé [CEREES], approval no. 1616698) and authorized by the French data protection authority (Commission Nationale de l’Informatique et des Libertés [CNIL], regulatory authorization DR-2020-311).

In accordance with French regulation, because patient data are exclusively from the SNDS, the processing of the data does not require further patient information or authorization.

#### Eligibility criteria

Eligibility criteria were women ≥18 years old who underwent a first MUS implantation identified with CCAM procedure codes (Supplemental 1). The index date was the date of the first MUS implantation. The observation period was from January 2011 to December 2019 and the inclusion period from January 2011 to December 2018 (allowing at least 1 year of follow-up). The data extraction period was from 2006 to 2019 to allow a look-back of at least 5 years for excluding patients who had a previous MUS implantation and to describe the characteristics of the population and identify potential confounding factors.

#### Treatment definition and assignment

Participants were classified at the index date according to the MUS implanted (i.e., TOT or TVT). Randomization was emulated by inverse probability weighting on a propensity score.

#### Outcomes and follow-up

The primary outcome was the cumulative incidence of a composite outcome: MUS removal or section. These surgical procedures were identified on hospital discharge summaries with procedure codes (Supplemental 1).

Secondary outcomes were the cumulative incidence of MUS removal and section (individual components of the composite outcome) and other severe complications (i.e., leading to hospitalization) also directly linked to MUS implantation: urinary retention, MUS erosion and infection, and cumulative incidence of at least one of these complications. We also considered reoperation for SUI by implanting a second MUS, as a proxy for efficacy. The above criteria were identified by ICD-10 or procedure codes (Supplemental 1). We considered other complications (chronic pain addressed by delivery of painkillers, overactive bladder addressed by delivery of antimuscarinic drugs, urinary tract infections addressed by a urine culture associated with delivery of urinary targeted antibiotics within 2 days before or 7 days after urine sampling), identified by ATC and biological codes, separately in the first year after implantation and thereafter (Supplemental 1). For urinary tract infection, the dual criterion (urine culture and urinary-targeted antibiotics delivery) allowed for identifying urinary infections with more specificity than with asymptomatic bacteriuria or non-urinary infection.

#### Covariates

We considered the calendar year of MUS implantation, age, the Charlson Comorbidity Index^12^ and low economic status (defined as being beneficiary of the “Complémentaire santé solidaire”, a health insurance benefit for people on very low incomes). Other covariates of interest (identified in the look-back period) included major comorbidities, identified by ICD-10 codes from hospitalization discharge data or LTD status, and ATC codes (to consider disease-specific drugs) (Supplemental 1). To describe the population and balance the two groups, we also considered as covariates of interest healthcare utilization during the previous year characterized by the number of outpatient visits, hospitalization, drugs delivered, medical care and specific urologic or gynecologic care, painkiller delivery, antimuscarinic drug delivery, antibiotic drug delivery, biological exams (e.g. urine culture, Complete Blood Count, C-reactive protein level, or pelvic floor imaging identified by procedure codes). These covariates were identified at the index date or in the look-back period.

#### Statistics

Baseline characteristics are described with median (interquartile range [IQR]) for continuous variables and frequency (%) for categorical variables.

Individuals were assigned to the treatment so that their observed data were compatible with the index treatment date (i.e., the first MUS implanted). They were then followed to the occurrence of the outcome of interest, death, or the end of the observation period, whatever came first.

Crude and weighted cumulative incidences of the outcomes were estimated by surgical approach (TVT or TOT) by using the Kaplan–Meier method. Incidence rates are reported at different times of interest with 95% confidence intervals (CIs). The causal contrast was the observational analogue of the intention-to-treat effect, and we estimated the average treatment effect in the whole population. To emulate the randomization, we used inverse probability of treatment weighting with a propensity score. The propensity score included covariates at the index date (MUS implantation): calendar year, age, Charlson Comorbidity Index, low economic status, LTD status, neurological disease (multiple sclerosis, spinal cord injury, Parkinson's disease, history of stroke) or diabetes, history of pelvic floor surgery, volume of use of healthcare the year before implantation (medical care and specific urologic or gynecologic care, painkiller delivery, antimuscarinic drug delivery, urinary-targeted antibiotic drug delivery, urine culture, pelvic floor imaging). Covariate balance between the two groups was assessed before and after weighting, and we considered an absolute standardized difference of <0.1 as evidence of balance (Supplemental 2).

The effect of TVT versus TOT on the censored outcomes of interest was assessed by using weighted Cox models (with TOT as the reference). Proportional-hazard assumption was assessed by inspecting the scaled Shoenfeld residuals.^13^ In case of deviation to the proportional-hazards assumption, the follow up was partitioned into several distinct intervals, within which the proportional hazards assumption was met. Parameters associated with each interval were introduced in the Cox model to account for the effect of time. To model the surgical approach on the amount of painkiller delivery, antimuscarinic drug delivery and urinary infections, we used a weighted Hurdle model, a two-part model that specifies one process for zero counts (using a logistic model) and another process for positive counts (using a negative binomial model to account for overdispersion) for analysing zero-inflated data, with an offset to account for follow-up duration.

Analyses involved using SAS Entreprise Guide 8.3 (SAS Institute) and R 4.2.1.

### Role of funding source

Funders did not have any role in study design, data collection, data analyses, interpretation nor writing of report.

## Results

**Fig. 1:**
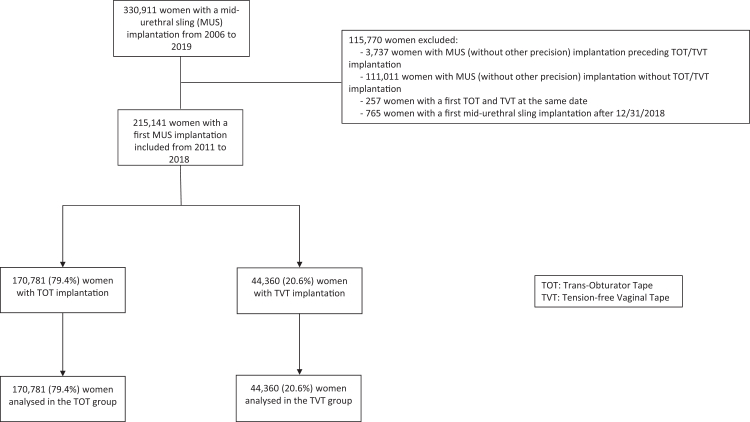
**Flow chart of the population**. MUS: mid-urethral sling; TVT: tension-free vaginal tape; TOT: trans-obturator tape.

In all, 215,141 patients underwent implantation (first implantation) from 2011 to 2018: 170,781 (79.4%) with TOT and 44,360 (20.6%) with TVT. The distribution of TOT and TVT implantations remained generally stable over the study period, with TOT procedures representing between 77.3% and 80.1% of all cases across the years. An exception was observed in 2011, during which the proportion of TOT implantations decreased to 69.7% (Supplemental 3). Fig. 1 shows the flow of participants in the study. The characteristics of the population at index date (implantation) before and after weighting are in Table 1. Median (IQR) age at implantation was 57 (47–68) years in the TOT group and 58 (48–69) years in the TVT group. Factors of poor outcomes (Parkinson's disease, diabetes, history of spinal cord injury and pelvic floor surgery) were more frequent in the TVT than TOT group. Median follow-up was 4.75 years (95% CI 4.74–4.77) in the TOT group and 5.09 years (95% CI 5.07–5.13) in the TVT group.

**Table 1:** Characteristics of women at implantation with mid-urethral sling (MUS) and in the propensity score-weighted population by MUS type: trans-obturator tape (TOT) or tension-free vaginal tape (TVT).

	Women with MUS implantation before propensity score weighting			Propensity score–weighted population with MUS		
	TOT (n = 170,781)	TVT (n = 44,360)	ASD	TOT	TVT	ASD
Median age (IQR)	57 (47–68)	58 (48–69)	0.0603	57 (48–68)	57 (47–68)	0.0009
Charlson Comorbidity Index^a^, N (%)						
0	63,241 (37.0%)	16,571 (37.4%)	0.0067	63,360 (37.1%)	16,434 (37.1%)	0.0002
1–2	69,929 (40.9%)	17,650 (39.8%)	0.0236	69,525 (40.7%)	18,028 (40.7%)	0.0004
3–4	17,301 (10.1%)	4559 (10.3%)	0.0048	17,352 (10.2%)	4496 (10.1%)	0.0004
≥5	20,310 (11.9%)	5580 (12.6%)	0.0209	20,558 (12.0%)	5351 (12.1%)	0.0012
Diabetes, N (%)	12,972 (7.6%)	3438 (7.8%)	0.0058	13,030 (7.6%)	3388 (7.6%)	0.0006
Multiple sclerosis, N (%)	473 (0.3%)	147 (0.3%)	0.0099	492 (0.3%)	128 (0.3%)	0.00007
Parkinson's disease, N (%)	700 (0.4%)	215 (0.5%)	0.0112	727 (0.4%)	189 (0.4%)	0.00009
History of spinal cord injury, N (%)	237 (0.1%)	123 (0.3%)	0.0304	286 (0.2%)	74 (0.2%)	0.0002
History of stroke, N (%)	3289 (1.9%)	935 (2.1%)	0.0129	3354 (2.0%)	877 (2.0%)	0.0011
History of pelvic floor surgery, N (%)	13,007 (7.6%)	3734 (8.4%)	0.0295	13,280 (7.8%)	3415 (7.7%)	0.0026
Median number of GP consultations during the year before implantation, (IQR)	7 (4–10)	7 (4–10)	0.0198	6 (4–10)	6 (4–10)	0.0010
Median number of urologic or gynecologic consultations during the year before implantation (IQR)	2 (2–3)	2 (2–3)	0.0421	2 (1–3)	2 (2–3)	0.0056
Patients with delivery of antibiotic drug boxes during the year before implantation, N (%)						
0 box	59,142 (34.6%)	14,850 (33.5%)	0.0244	58,724 (34.4%)	15,169 (34.2%)	0.0031
1–2 boxes	46,137 (27.0%)	11,992 (27.0%)	0.0004	46,156 (27.0%)	12,010 (27.1%)	0.0018
3–6 boxes	43,130 (25.3%)	11,270 (25.4%)	0.0035	43,187 (25.3%)	11,212 (25.3%)	0.0004
≥7 boxes	22,372 (13.1%)	6248 (14.1%)	0.0287	22,728 (13.3%)	5918 (13.4%)	0.0014
Patients with delivery of antimuscarinic drug boxes during the year before implantation, N (%)						
0 box	141,190 (82.7%)	35,662 (80.4%)	0.0588	140,382 (82.2%)	36,372 (82.1%)	0.0028
1 box	9344 (5.5%)	2713 (6.1%)	0.0276	9580 (5.6%)	2506 (5.7%)	0.0020
2–6 boxes	13,767 (8.1%)	4046 (9.1%)	0.0378	14,150 (8.3%)	3693 (8.3%)	0.0018
≥7 boxes	6480 (3.8%)	1939 (4.4%)	0.0291	6684 (3.9%)	1739 (3.9%)	0.0006
Patients with delivery of painkiller boxes during the year before implantation, N (%)						
0 box	35,816 (21.0%)	9628 (21.7%)	0.0179	36,089 (21.1%)	9406 (21.2%)	0.0024
1–3 boxes	33,357 (19.5%)	8484 (19.1%)	0.0103	33,216 (19.4%)	8617 (19.4%)	0.00002
4–20 boxes	68,441 (40.1%)	17,299 (39.0%)	0.0221	68,057 (39.8%)	17,623 (39.8%)	0.0015
≥21 boxes	33,167 (19.4%)	8949 (20.2%)	0.0189	33,432 (19.6%)	8664 (19.6%)	0.0005
Patients with investigations of CBC during the year before implantation, N (%)						
0 test	26,421 (15.5%)	6088 (13.7%)	0.0495	25,798 (15.1%)	6655 (15.0%)	0.0024
1–2 tests	10,7547 (63.0%)	28,162 (63.5%)	0.0106	10,7749 (63.1%)	28,000 (63.2%)	0.0021
≥3 tests	36,813 (21.6%)	10,110 (22.8%)	0.0297	37,247 (21.8%)	9655 (21.8%)	0.0005
Patients with investigations of CRP level during the year before implantation, N (%)						
0 test	98,603 (57.7%)	25,353 (57.2%)	0.0118	98,400 (57.6%)	25,511 (57.6%)	0.0008
1–2 tests	62,268 (36.5%)	16,265 (36.7%)	0.0043	62,352 (36.5%)	16,196 (36.6%)	0.0009
≥3 tests	9910 (5.8%)	2742 (6.2%)	0.0159	10,043 (5.9%)	2603 (5.9%)	0.0003
Patients with investigations of urine culture during the year before implantation, N (%)						
0 test	38,326 (22.4%)	9187 (20.7%)	0.0421	37,710 (22.1%)	9738 (22.0%)	0.0024
1–2 tests	94,219 (55.2%)	24,482 (55.2%)	0.0004	94,238 (55.2%)	24,475 (55.2%)	0.0012
≥3 tests	38,236 (22.4%)	10,691 (24.1%)	0.0405	38,847 (22.7%)	10,097 (22.8%)	0.0010
Patients with investigations by abdominal ultrasonography during the previous year, N (%)	1449 (0.8%)	354 (0.8%)	0.0056	1430 (0.8%)	366 (0.8%)	0.0013
Patients with investigations by abdominal CT scan during the previous year, N (%)	4418 (2.6%)	1199 (2.7%)	0.0072	4459 (2.6%)	1158 (2.6%)	0.0001
Patients with investigations of abdominal MRI during the previous year, N (%)	2212 (1.3%)	557 (1.3%)	0.0035	2198 (1.3%)	572 (1.3%)	0.0003
Status of the hospital where MUS was implanted, N (%)						
Public	68,554 (40.1%)	19,548 (44.1%)	0.0796	69,990 (41.0%)	18,350 (41.4%)	0.0088
Private	102,227 (59.9%)	24,812 (55.9%)	0.0796	100,805 (59.0%)	25,959 (58.6%)	0.0088
Low economic status, N (%)	7186 (4.2%)	1801 (4.1%)	0.0074	7132 (4.2%)	1841 (4.2%)	0.0010

ASD: absolute standardized difference; IQR: interquartile range; GP: general practitioner; CBC: complete blood count; CRP: C-reactive protein.

a Not available for all patients.

Table 2 summarizes both the specifications of the target trial and its emulation with observational data.

**Table 2:** Specification of the target trial and its emulation.

	Target trial specification	Target trial emulation
Eligibility criteria	Inclusion criteria: ⁃ Adult women (≥18 yo) ⁃ Scheduled for MUS implantation for stress urinary incontinence (first implantation).	Inclusion criteria: ⁃ Adult women (≥18 yo) ⁃ MUS implantation for stress urinary incontinence (first implantation).
Treatment strategies	Implantation of MUS whether: ⁃ TVT: retropubic approach or ⁃ TOT: transobturator approach.	Same as for the target trial.
Assignment to treatment strategies	Participants are randomly assigned to TVT or TOT Participants and their treating physicians are aware of the assigned treatment strategy.	Participants are classified at baseline accorded to the MUS implanted. Randomization is emulated by inverse probability weighting on a propensity score.
Outcomes	The primary outcome is the cumulative incidence of surgical procedure related to MUS: MUS removal or section (composite outcome). Secondary outcomes are the cumulative incidence of MUS removal and section, hospitalization for urinary retention, erosion, infection or of at least one of these five outcomes, and a second MUS implantation, as a proxy for efficacy, such as the number of pads used in 24 h and the resolution of incontinence (patients-reported outcomes). Other complications (chronic pain, overactive bladder, urinary tract infection) are also considered, separately in the first year after implantation, and thereafter.	Same as for the target trial except for the number of pads used in 24 h and the resolution of incontinence (patient-reported outcomes); data unavailable in the database. Chronic pain is evaluated by painkiller delivery, overactive bladder is evaluated by antimuscarinic drug delivery and urinary tract infection is evaluated by a urine culture associated with the delivery of urinary targeted antibiotics within 2 days before or 7 days after urine sampling.
Follow-up	Patients are followed from randomization to the occurrence of the outcome of interest, death, lost to follow-up or the end of the follow-up period (at least 1 year of follow-up, up to 10 years after randomization).	Patients are followed from the index date (MUS implantation) to the occurrence of the outcome of interest, death, or the end of the observation period, whatever comes first (at least 1 year of follow-up, up to 9 years).
Causal contrast	Intention-to-treat effect.	Observational analogue of intention-to-treat effect.
Analysis	Cox model.	Weighted Cox model (Inverse Probability Weighting on a propensity score).

MUS: mid-urethral sling; TVT: tension-free vaginal tape; TOT: trans-obturator tape.

### Primary outcome

**Fig. 2:**
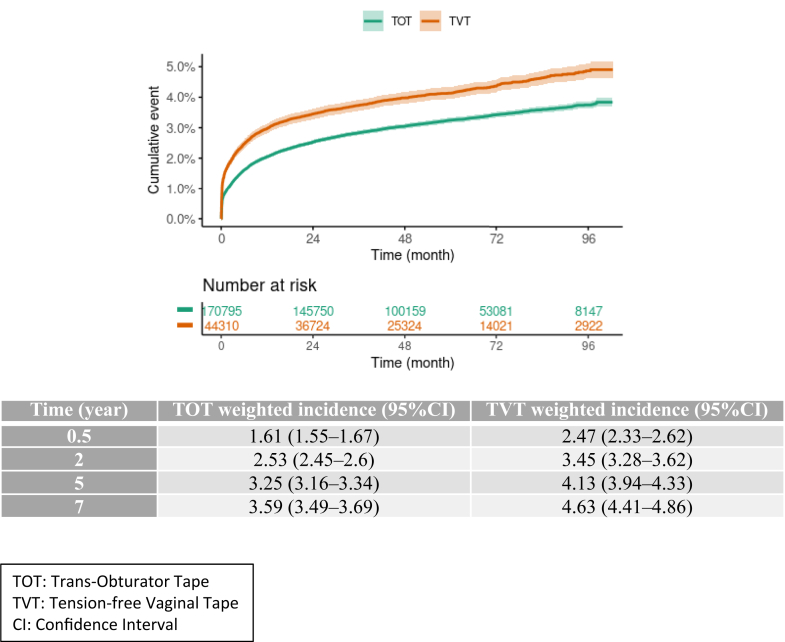
**Weighted cumulative incidence of mid-urethral sling (MUS) removal or sectio**n. The dark line represents the cumulative incidence, and the lighter area the 95% confidence interval. TVT: tension-free vaginal tape; TOT: trans-obturator tape; CI: confidence interval.

The crude and weighted cumulative incidences of reoperation for MUS removal or section were higher in TVT than TOT group (Fig. 2 and Supplemental 4). At 5 years, the weighted cumulative incidence of MUS removal or section was 3.25 (95% CI 3.16–3.34) in the TOT group and 4.13 (95% CI 3.94–4.33) in the TVT group. At 7 years, it was 3.59 (95% CI 3.49–3.69) and 4.63 (95% CI 4.41–4.86), respectively.

In the weighted analysis, to account for the time-dependent effect, several HRs are reported indicating very early, early or late effects. The risk of MUS removal or section was higher in the TVT than TOT group (p < 0.001), higher in the very short-term ([0–3 months] after implantation [HR 1.65, 95% CI 1.52–1.78]) than short-term ([3–12 months] after implantation [HR 1.19, 95% CI 1.06–1.32]) and mid-term ([1–5 years] after implantation [HR 0.98, 95% CI 0.88–1.1]) and re-increased in the long-term (≥5 years after implantation [HR 1.44, 95% CI 1.13–1.85]) (Table 3).

**Table 3:** Effect of the surgical approach, tension-free vaginal tape (TVT) versus trans-obturator tape (TOT), on surgical outcomes in the weighted population, estimated with a Cox model.

	Time from implantation	HR (95% CI)	P value
Primary outcome			
MUS removal or section			
	[0–3 months]	1.65 (1.52–1.78)	<0.001
	[3 months–1 year]	1.19 (1.06–1.32)	
	[1 year–5 years]	0.98 (0.88–1.1)	
	≥5 years	1.44 (1.13–1.85)	
Secondary outcomes			
MUS section			
	[0–3 months]	1.77 (1.6–1.96)	<0.001
	[3 months–1 year]	1.12 (0.93–1.34)	
	[1 year–5 years]	1.07 (0.88–1.3)	
	≥5 years	1.38 (0.8–2.37)	
MUS removal			
	[0–3 months]	1.47 (1.3–1.67)	<0.001
	[3 months–1 year]	1.22 (1.06–1.4)	
	[1 year–5 years]	0.94 (0.82–1.07)	
	≥5 years	1.45 (1.1–1.92)	
Second MUS implantation			
	[0–3 months]	0.887 (0.729–1.078)	<0.001
	[3 months–1 year]	0.767 (0.677–0.868)	
	[1 year–3 years]	0.806 (0.706–0.919)	
	≥3 years	0.894 (0.764–1.047)	
Hospitalization for urinary retention			
	[0–3 months]	1.76 (1.63–1.9)	<0.001
	[3 months–1 year]	1.2 (1.06–1.36)	
	[1 year–3 years]	1.13 (1.01–1.25)	
	≥3 years	1.08 (0.97–1.2)	
Hospitalization for MUS infection			
	[0–6 months]	1.48 (1.16–1.88)	0.005
	[6 months–2 years]	1.11 (0.87–1.41)	
	[2 years–5 years]	0.83 (0.65–1.07)	
	≥5 years	1.43 (1.02–1.99)	
Hospitalization for MUS erosion			
	[0–3 months]	1.61 (1.42–1.83)	<0.001
	[3 months–1 year]	1.37 (1.2–1.57)	
	[1 year–5 years]	1.05 (0.94–1.19)	
	≥5 years	1.5 (1.18–1.91)	

MUS: mid-urethral sling, CI: confidence interval.

TOT is the group of reference.

### Secondary outcomes

**Fig. 3:**
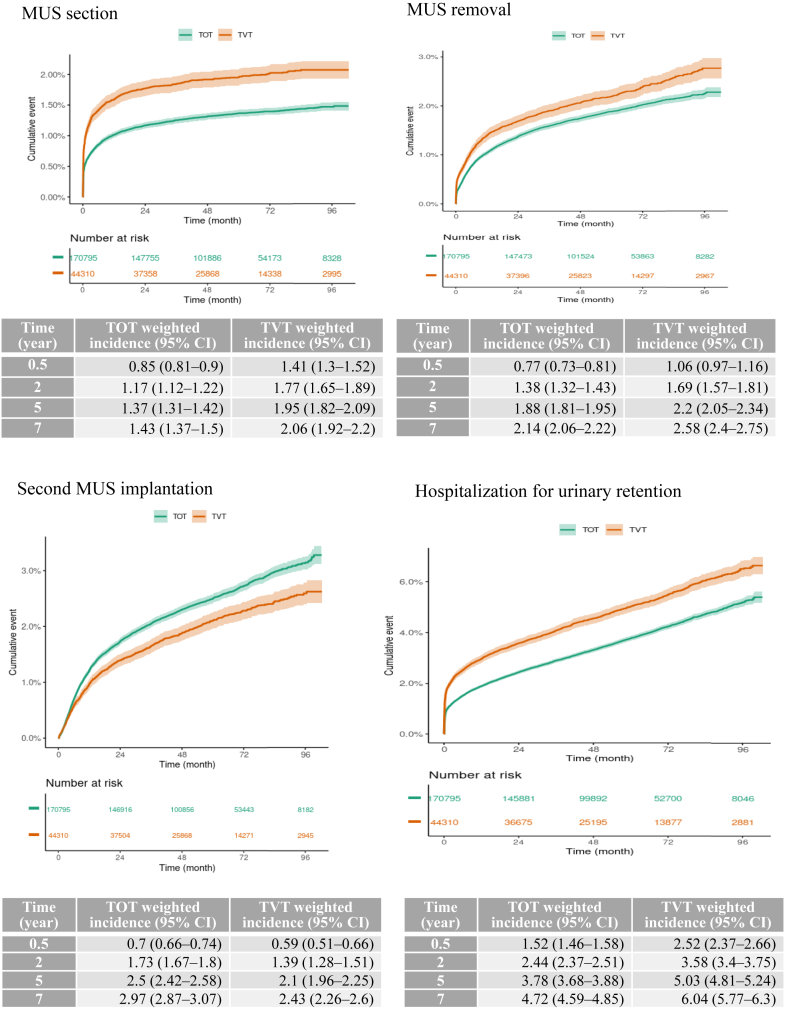

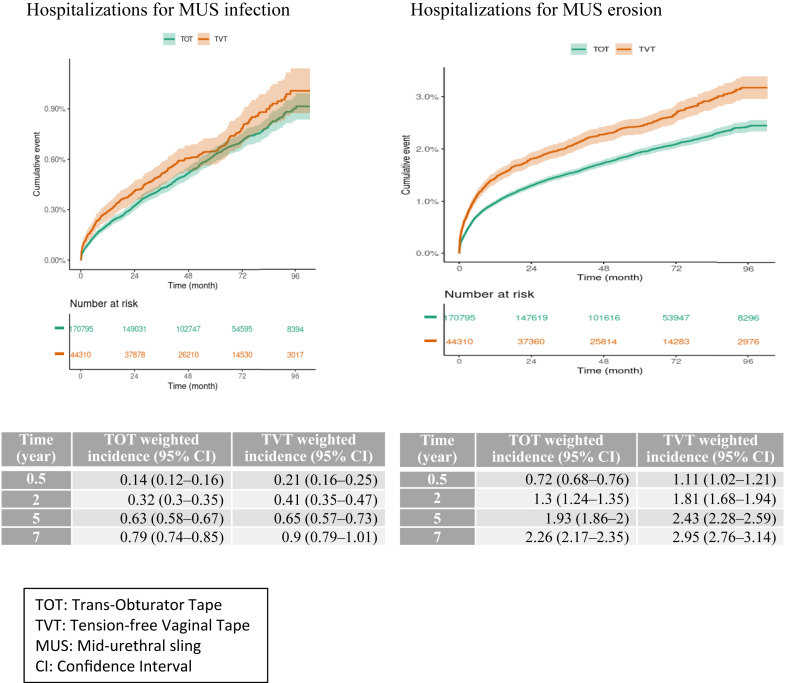
**Weighted cumulative incidence of secondary outcomes**. The dark line represents the cumulative incidence, and the lighter area the 95% confidence interval. MUS: mid-urethral sling; TVT: tension-free vaginal tape; TOT: trans-obturator tape; CI: confidence interval.

**Fig. 4:**
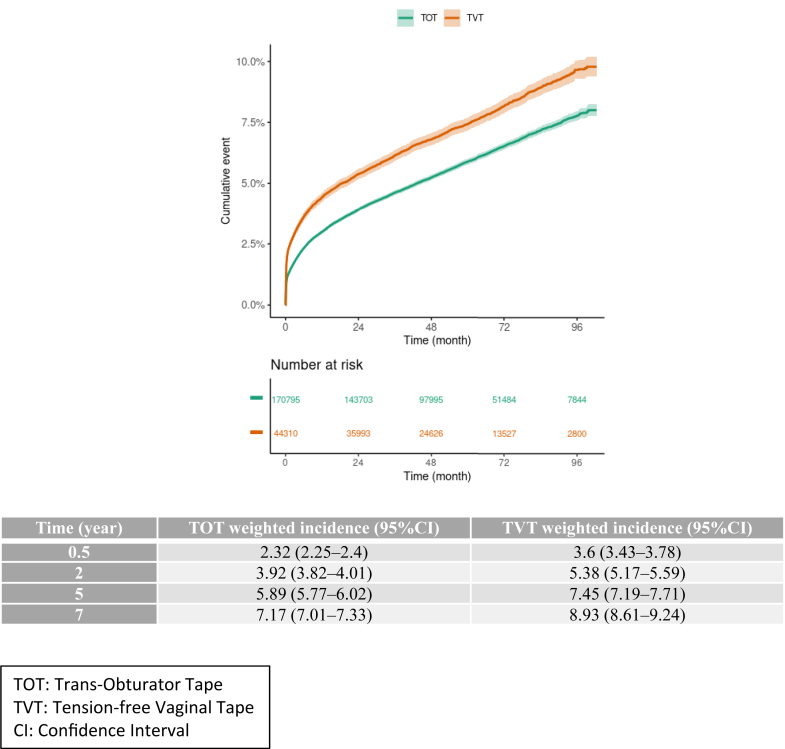
**Weighted cumulative incidence of at least one severe complication after mid-urethral sling (MUS) implantation**. The dark line represents the cumulative incidence, and the lighter area the 95% confidence interval. TVT: tension-free vaginal tape; TOT: trans-obturator tape; CI: confidence interval.

Fig. 3 shows the cumulative incidences of the secondary outcomes in the weighted population. Crude cumulative incidences are reported in Supplemental 5. Over time, in the weighted population, the risk of MUS removal and section and hospitalization for urinary retention and MUS erosion and infection was significantly greater in the TVT than TOT group (Table 3), with also a time-varying effect. The crude and weighted cumulative incidences of at least one of these complications were significantly greater in the TVT than TOT group (Supplemental 6 and Fig. 4, respectively**)**.

However, there were more second MUS implantations in the TOT than TVT group. The risks of other complications (painkiller delivery, antimuscarinic drug delivery and urinary infections) are reported in Table 4. The MUS groups did not differ in painkiller delivery (at least one drug delivery) in the first year after implantation, but at ≥ 1 year after implantation, painkiller delivery was less frequent in the TVT than TOT group, with no significant difference in number of boxes of painkillers delivered among the patients with at least one delivery. The MUS groups did not differ in antimuscarinic drug delivery. Finally, urinary tract infections were more frequent in the TVT than TOT group during and after 1 year after implantation, both in terms of patients with at least one infection and number of infections per patient with at least one infection.

**Table 4:** Effect of the surgical approach, tension-free vaginal tape (TVT) versus trans-obturator tape (TOT), on drug outcomes in the weighted population, estimated with a Hurdle model.

	OR (95% CI)	IRR (95% CI)	P value (global)
**Pain killer delivery**			
Since implantation	0.98 (0.92–1.04)	1.01 (0.99–1.02)	0.40
The first year after implantation	1 (0.97–1.03)	1.01 (0.99–1.03)	0.44
After the first year after implantation	0.92 (0.89–0.94)	1.01 (0.99–1.03)	<0.0001
**Antimuscarinic drug delivery**			
Since implantation	1.01 (0.99–1.04)	0.98 (0.93–1.02)	0.45
The first year after implantation	1.01 (0.98–1.04)	0.98 (0.94–1.01)	0.40
After the first year after implantation	0.99 (0.96–1.02)	0.98 (0.93–1.04)	0.74
**Urinary infections**			
Since implantation	1.09 (1.07–1.11)	1.07 (1.03–1.1)	<0.0001
The first year after implantation	1.14 (1.11–1.16)	1.07 (1.03–1.11)	<0.0001
After the first year after implantation	1.04 (1.02–1.06)	1.05 (1.01–1.09)	<0.0001

OR: odds ratio; CI: confidence interval; IRR Incidence Rate Ratio (by person-month).

TOT is the group of reference.

Pain killer delivery is a proxy to estimate chronic pain.

Antimuscarinic drug delivery is a proxy to estimate overactive bladder.

Urinary tract infections are addressed by a urine culture in the 2 days before or 7 days after the delivery of related urinary-targeted antibiotics.

The Hurdle model is a two-part model that specifies one process for zero counts versus non zero counts (e.g. no pain killer delivery versus at least one) which result is expressed with an OR, and another process for non zero counts (e.g. those with at least one pain killer delivery) comparing the number of counts (e.g. the number of boxes of pain killer delivered) which result is expressed with an IRR.

## Discussion

Using French national healthcare data (SNDS), we emulated a target randomized trial comparing TOT and TVT approaches to SUI in 215,141 women who underwent mid-urethral MUS implantation, with observational data in the real world setting and long-term follow-up. The cumulative incidence of MUS removal or section was higher in the TVT than TOT group, 4.13% versus 3.25% at 5 years and 4.63% versus 3.59% at 7 years. Also, the risk of other safety criteria (MUS removal and section and hospitalization for urinary retention, MUS erosion and infection) was greater in the TVT than TOT group. Finally, the risk of urinary tract infection was increased 14% in the TVT group (for individuals with at least one infection) during the first year, with a higher frequency among those affected, and increased 4% beyond 12 months as compared with the TOT group. In contrast, the risk of painkiller use was decreased by 8% in the TVT group after the first year (no significant difference before), with no change in quantity delivered to users. However, second MUS implantation was more frequent in the TOT than TVT group, which may suggest better effectiveness of the TVT approach. This result should be interpreted with caution because it relies on only SUI leading to reoperation (small proportion of recurrence or persistent SUI) and this was a secondary outcome.

In the literature, seven randomized controlled trials had compared TOT and TVT.^14^, ^15^, ^16^, ^17^, ^18^, ^19^, ^20^ The reintervention rates ranged from 2% to 3% for TOT and 2%–7% for TVT at 5 years. These rates are of the same order of magnitude as those we report. These studies found no significant difference between TOT and TVT approaches in terms of safety (reoperation or complications) or effectiveness (continence rate, patient-reported outcomes). However, they were too small (from 72 to 597 patients included) to compare the incidence of a rather rare event with sufficient power and with a high percentage of lost to follow-up (up to 40%), as reported in Supplemental 7, and thus prone to attrition bias. Clinical trials are often not designed with sufficient size to effectively analyse rare events. Moreover, these clinical trials were conducted in expert centres with experienced practitioners. Furthermore, in trials, participants are selected with restricted eligibility criteria and in general differ from patients in real life.

These data are also consistent with those from the VIGI-MESH register, which prospectively records severe complications of MUS of 2682 participants in 27 French centres. Started in 2017, the cumulative 2-year estimate of serious complications was 2.9% (95% CI 1.9–4.3) in the TOT group and 5.8% (95% CI 4.8–7.0) in the TVT group after weighting with a propensity score (including age, body mass index and major comorbidities).^21^ This finding is consistent with the cumulative incidence of at least one complication at 2 years in our study: 3.92% (95% CI 3.82–4.01) for TOT and 5.38% (95% CI 5.17–5.59) for TVT. However, the previous study reported that 852 patients (31.8%) underwent TOT implantation and 1830 (68.3%) TVT, whereas we reported 79.4% TOT implantation and 20.6% TVT in real-world practice. This finding raises the question of selection bias: the centres included in VIGI-MESH are more expert and their surgical practices differ from those of all centres in France, with a preference for TVT implantation. Nevertheless, the TOT/TVT ratio observed in this study is the reverse of what is typically reported in many other countries, where TVT remains the dominant approach. TOT has been more widely adopted in France, especially by gynecologists, who make up a substantial proportion of surgeons performing MUS, because most of them do not perform cystoscopy.

Our study has several strengths. This is the first study reporting real-life data on long-term complications of MUS. Second, the population included is quasi-exhaustive in that the SNDS covers 99% of the population living in France and all centres implanting MUS in France were considered, so external validity was excellent. Moreover, our large population, with a long follow-up and no lost to follow-up, provides sufficient statistical power to analyse differences in rare events. Third, we used the methodological framework of emulated trials in order to minimize the risk of bias in the context of observational data. The target trial emulation framework was first described in 2016 by Hernan and Robins; it aims to limit bias in observational studies and improve transparency with regard to the precise scientific question investigated.^22^^,^^23^ Finally, safety data in general and reintervention data in particular are rarely reported beyond 2 years and are heterogeneous.^7^

The study also has limitations. First, the medico-administrative nature of the data misses deep clinical and urodynamic characteristics and complications. Therefore, we cannot exclude some imbalance in the characteristics at baseline, and some data (such as painkiller delivery) are not specific. Moreover, the level of detail in the SNDS does not extend to identifying the specific brand or model of the sling, nor does it allow us to differentiate between the inside-out and outside-in techniques for TOT, as such distinctions are not captured in the coding system. However, it is unlikely that this would significantly impact the effectiveness or safety of device implantation, as the different brands of MUS share the same material and are implanted with the same surgical technique. Only the implantation instrument may be a little different depending on the MUS. Second, there was a lack of efficacy data (except for reoperation for SUI, which can be considered a proxy for efficacy), thereby precluding the assessment of the risk–benefit balance. Third, we reported data up to 2019 only, but there were no modifications in clinical practice in SUI treatment after that. Finally, this was an observational study, so we cannot rule out residual confounding.

These results may have an important impact on clinical practice. They should be reported in the information sheet for patients before MUS implantation in terms of risk of reoperation and other complications. We have shown that MUS complications may occur even more than 5 years after implantation, so patients should be followed in the long term after implantation. In addition, these results could call into question the surgical approach to MUS implantation, favouring the TOT approach for a first implantation. This decision must be balanced with the type of complications patients may experience, focusing not only on reoperation rate (more frequent with TVT than TOT) but also painkiller use (more frequent with TOT than TVT) and urinary infections (more frequent with TVT than TOT). Finally, centralizing SUI treatments in expert, high-volume centers could be beneficial for patient outcomes; this approach is supported by a recent French decree, which requires a minimum annual number of urinary incontinence procedures per center to ensure adequate experience and quality of care.^24^ However, the two-year safety outcomes reported in the VIGI-MESH study, despite being conducted in expert centers, are comparable to those observed in our nationwide real-world data, suggesting that similar safety levels can be achieved across diverse healthcare settings in France.

In conclusion, we report real-life long-term safety data from all synthetic MUS implantations, TVT or TOT, performed in France from 2011 to 2018 for managing female SUI. Reinterventions (sling removal or section) could occur even more than 5 years after implantation and were more frequent with TVT than TOT. Furthermore, MUS removal and section, hospitalization for urinary retention and MUS erosion and infection, as well as urinary infections were more frequent after TVT than TOT, but use of painkillers after 1 year and second MUS implantation for SUI recurrence were less frequent with TVT.

## Contributors

The conceptualization of the study was carried out by CGT, PM, ECK and FT. Funding acquisition was led by CGT, PM, ECK and FT. Methodology was developed by CGT, SG, YDR and FT. Formal analysis was conducted by MHTDT, YDR and FT. SG and MHTDT have checked the underlying data. Validation was ensured by CGT, MHTDT, AD, ECK, FT. FT served as project administrator and supervisor. The original draft was written by CGT. SG, MHTDT, YDR, AD, PM, ECK and FT provided critical reviews and editing of the manuscript.

All authors read and approved the final version of the manuscript.

## Data sharing statement

Due to the nature of the research and to privacy regulations, raw data cannot be shared.

## Declaration of interests

PM is chief medical officer at UroMems. ECK is consultant for Boston Scientific, Uromedica and UroMems.

## Appendix A Supplementary data

Supplementals

## References

[1] ICS (2015). International continence society fact sheets. https://www.ics.org/public/factsheets.

[2] Delorme E. (2001). [Transobturator urethral suspension: mini-invasive procedure in the treatment of stress urinary incontinence in women]. Prog Urol.

[3] Nambiar A.K., Arlandis S., Bø K. (2022). European association of urology guidelines on the diagnosis and management of female non-neurogenic lower urinary tract symptoms. Part 1: diagnostics, overactive bladder, stress urinary incontinence, and mixed urinary incontinence. Eur Urol.

[4] Frati A., Poncelet C., Madelenat P., Luton D., Ducarme G. (2009). [Evolution of surgical operations for female for stress urinary incontinence in gynaecology departments of Parisian public hospitals between 2002 and 2006]. Gynecol Obstet Fertil.

[5] Ford A.A., Rogerson L., Cody J.D., Aluko P., Ogah J.A. (2017). Mid-urethral sling operations for stress urinary incontinence in women. Cochrane Database Syst Rev.

[6] Gurol-Urganci I., Geary R.S., Mamza J.B. (2018). Long-term rate of mesh sling removal following midurethral mesh sling insertion among women with stress urinary incontinence. JAMA.

[7] Guillot-Tantay C., Van Kerrebroeck P., Chartier-Kastler E., Dechartres A., Tubach F. (2023). Long-term safety of synthetic midurethral sling implantation for the treatment of stress urinary incontinence in adult women: a systematic review. Eur Urol Open Sci.

[8] Keltie K., Elneil S., Monga A. (2017). Complications following vaginal mesh procedures for stress urinary incontinence: an 8 year study of 92,246 women. Sci Rep.

[9] Davey M. (2022). Johnson & Johnson reaches $300m settlement over pelvic mesh implants. https://www.theguardian.com/business/2022/sep/12/johnson-johnson-reaches-300m-settlement-over-pelvic-mesh-implants.

[10] NICE guideline (2019).

[11] Tuppin P., Rudant J., Constantinou P. (2017). Value of a national administrative database to guide public decisions: From the système national d’information interrégimes de l'Assurance Maladie (SNIIRAM) to the système national des données de santé (SNDS) in France. Rev Epidemiol Sante Publique.

[12] Bannay A., Chaignot C., Blotière P.-O. (2016). The best use of the Charlson comorbidity index with electronic health care database to predict mortality. Med Care.

[13] Grambsch P.M., Therneau T.M. (1994). Proportional hazards tests and diagnostics based on weighted residuals. Biometrika.

[14] Laurikainen E., Valpas A., Aukee P. (2014). Five-year results of a randomized trial comparing retropubic and transobturator midurethral slings for stress incontinence. Eur Urol.

[15] Tammaa A., Aigmüller T., Hanzal E. (2018). Retropubic versus transobturator tension-free vaginal tape (TVT vs TVT-O): five-year results of the Austrian randomized trial. Neurourol Urodyn.

[16] Zhang Z., Zhu L., Xu T., Lang J. (2016). Retropubic tension-free vaginal tape and inside-out transobturator tape: a long-term randomized trial. Int Urogynecol J.

[17] Angioli R., Plotti F., Muzii L., Montera R., Panici P.B., Zullo M. (2010). Tension-free vaginal tape versus transobturator suburethral tape: five-year follow-up results of a prospective, randomised trial. Eur Urol.

[18] Salo H., Sova H., Laru J. (2023). Long-term results of a prospective randomized trial comparing tension-free vaginal tape versus transobturator tape in stress urinary incontinence. Int Urogynecol J.

[19] Kenton K., Stoddard A.M., Zyczynski H. (2015). 5-year longitudinal followup after retropubic and transobturator mid urethral slings. J Urol.

[20] Ross S., Tang S., Eliasziw M. (2016). Transobturator tape versus retropubic tension-free vaginal tape for stress urinary incontinence: 5-year safety and effectiveness outcomes following a randomised trial. Int Urogynecol J.

[21] Armengaud C., Fauconnier A., Drioueche H. (2024). Serious complications and recurrences after retropubic vs transobturator midurethral sling procedures for 2682 patients in the VIGI-MESH register. Am J Obstet Gynecol.

[22] Hernán M.A., Sauer B.C., Hernández-Díaz S., Platt R., Shrier I. (2016). Specifying a target trial prevents immortal time bias and other self-inflicted injuries in observational analyses. J Clin Epidemiol.

[23] Hernán M.A., Robins J.M. (2016). Using big data to emulate a target trial when a randomized trial is not available. Am J Epidemiol.

[24] Ministre du travail, de la santé, des solidarités et des familles (2025). Arrêté du 25 avril 2025 encadrant la pratique des actes d’implantation associés à la pose de bandelettes sous-urétrales destinés au traitement chirurgical de l’incontinence urinaire d’effort chez la femme en application des dispositions de l’article L. 1151-1 du code de la santé publique. https://www.legifrance.gouv.fr/jorf/id/JORFTEXT000051533063.

