# Supplementary material for: Long-term safety of mid-urethral sling for stress urinary incontinence in women: an emulated trial using French national health data system
Source: eClinicalMedicine. 2025 Sep 15;87:103411. doi: 10.1016/j.eclinm.2025.103411 (PMC12496164; doi:10.1016/j.eclinm.2025.103411)
Supplement: Supplementals [file mmc1.pptx]

## Slide 1
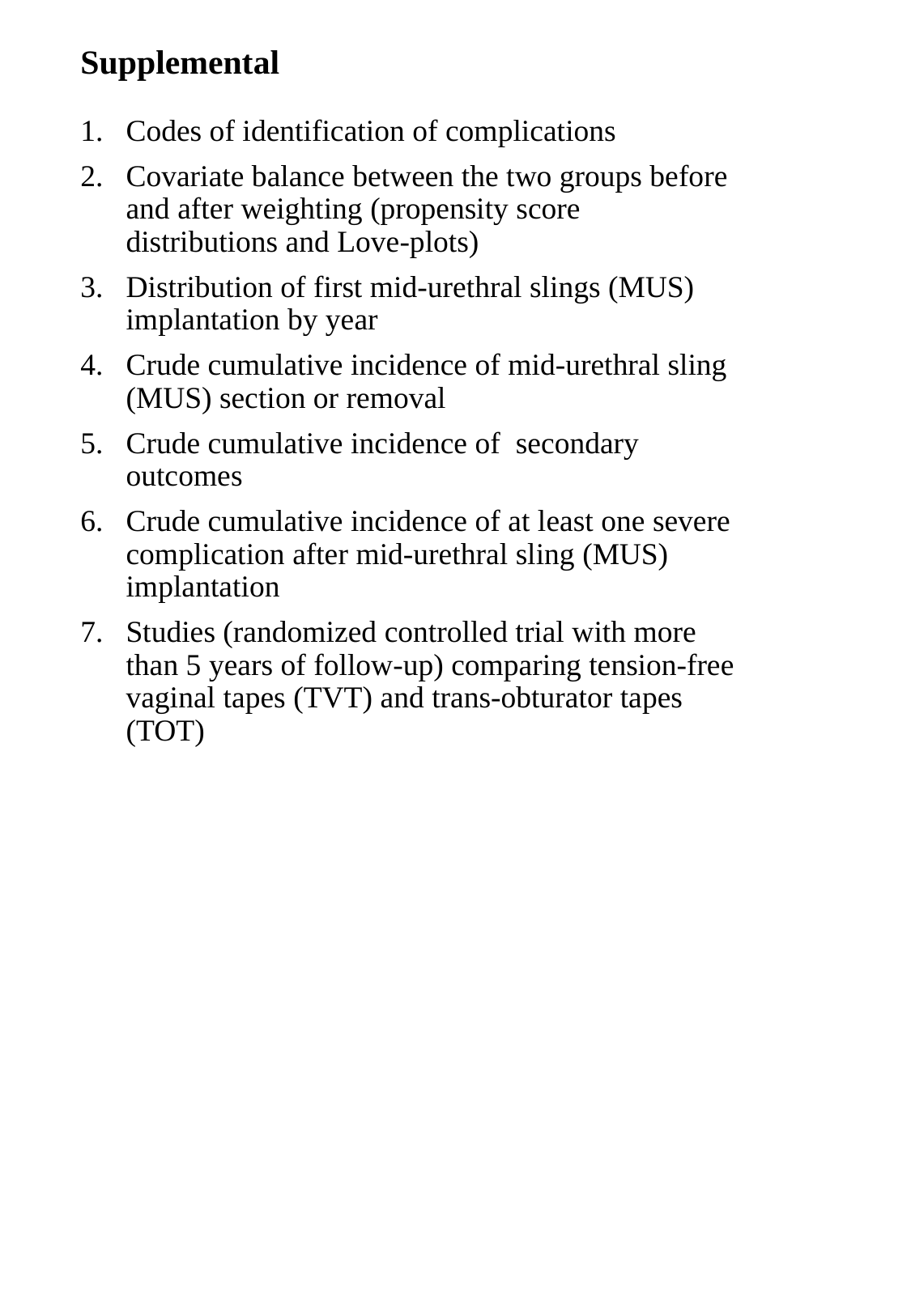

# Supplemental
Codes of identification of complications
Covariate balance between the two groups before and after weighting (propensity score distributions and Love-plots)
Distribution of first mid-urethral slings (MUS) implantation by year
Crude cumulative incidence of mid-urethral sling (MUS) section or removal
Crude cumulative incidence of secondary outcomes
Crude cumulative incidence of at least one severe complication after mid-urethral sling (MUS) implantation
Studies (randomized controlled trial with more than 5 years of follow-up) comparing tension-free vaginal tapes (TVT) and trans-obturator tapes (TOT)

## Slide 2
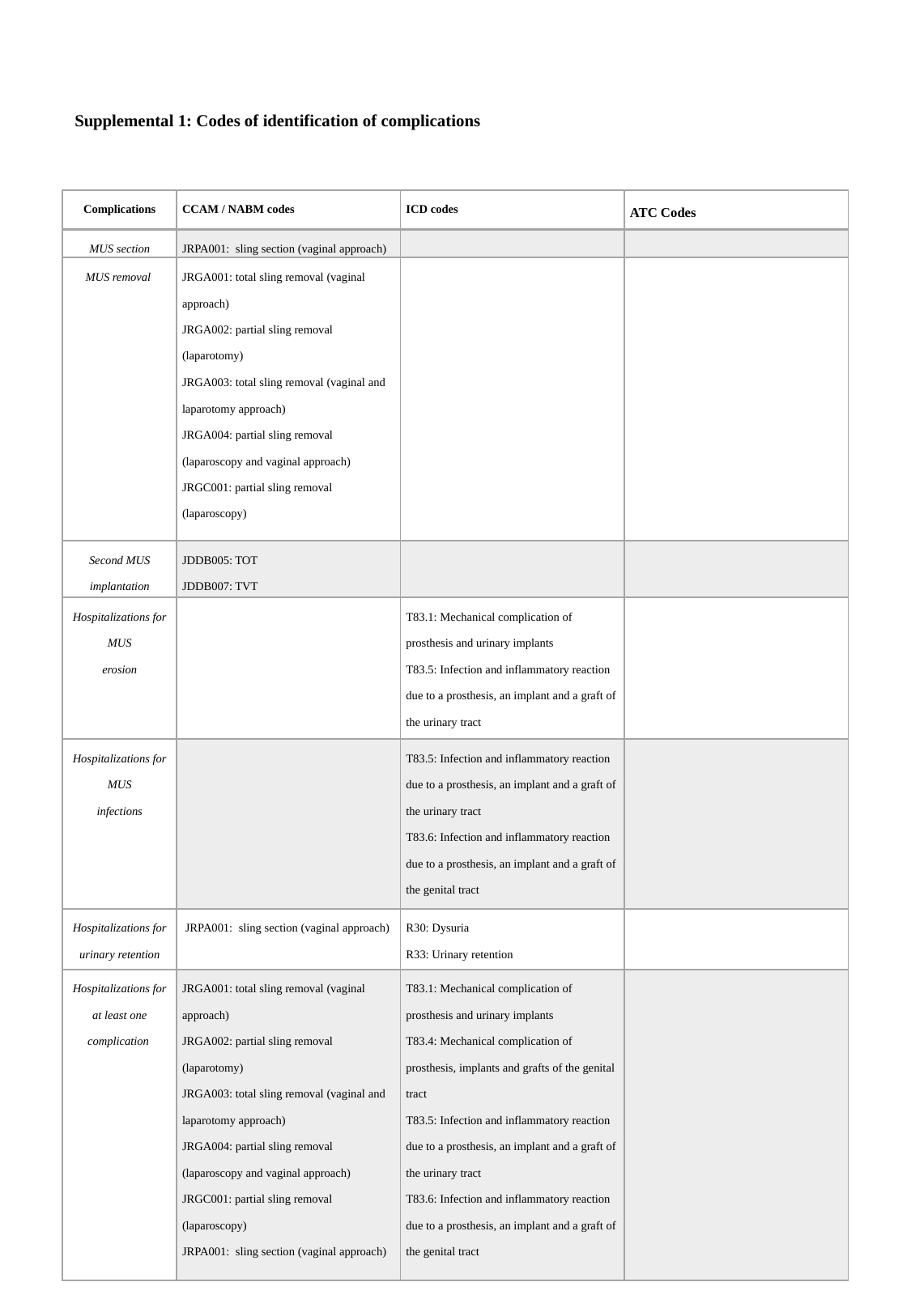

# Supplemental 1: Codes of identification of complications
| Complications | CCAM / NABM codes | ICD codes | ATC Codes |
| --- | --- | --- | --- |
| MUS section | JRPA001: sling section (vaginal approach) | | |
| MUS removal | JRGA001: total sling removal (vaginal approach) JRGA002: partial sling removal (laparotomy) JRGA003: total sling removal (vaginal and laparotomy approach) JRGA004: partial sling removal (laparoscopy and vaginal approach) JRGC001: partial sling removal (laparoscopy) | | |
| Second MUS implantation | JDDB005: TOT JDDB007: TVT | | |
| Hospitalizations for MUS erosion | | T83.1: Mechanical complication of prosthesis and urinary implants T83.5: Infection and inflammatory reaction due to a prosthesis, an implant and a graft of the urinary tract | |
| Hospitalizations for MUS infections | | T83.5: Infection and inflammatory reaction due to a prosthesis, an implant and a graft of the urinary tract T83.6: Infection and inflammatory reaction due to a prosthesis, an implant and a graft of the genital tract | |
| Hospitalizations for urinary retention | JRPA001: sling section (vaginal approach) | R30: Dysuria R33: Urinary retention | |
| Hospitalizations for at least one complication | JRGA001: total sling removal (vaginal approach) JRGA002: partial sling removal (laparotomy) JRGA003: total sling removal (vaginal and laparotomy approach) JRGA004: partial sling removal (laparoscopy and vaginal approach) JRGC001: partial sling removal (laparoscopy) JRPA001: sling section (vaginal approach) | T83.1: Mechanical complication of prosthesis and urinary implants T83.4: Mechanical complication of prosthesis, implants and grafts of the genital tract T83.5: Infection and inflammatory reaction due to a prosthesis, an implant and a graft of the urinary tract T83.6: Infection and inflammatory reaction due to a prosthesis, an implant and a graft of the genital tract | |

## Slide 3
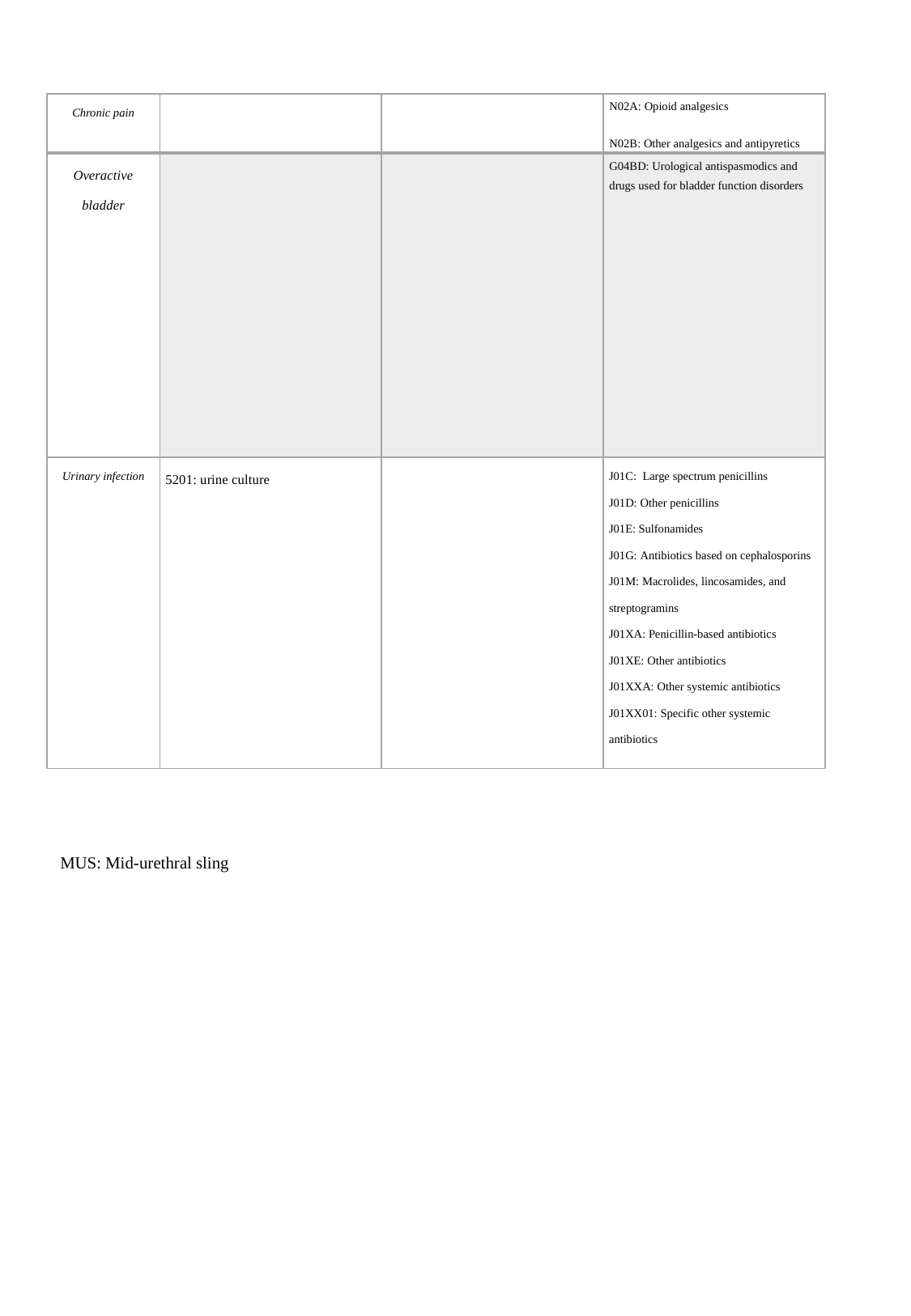

| Chronic pain | | | N02A: Opioid analgesics N02B: Other analgesics and antipyretics |
| --- | --- | --- | --- |
| Overactive bladder | | | G04BD: Urological antispasmodics and drugs used for bladder function disorders |
| Urinary infection | 5201: urine culture | | J01C: Large spectrum penicillins J01D: Other penicillins J01E: Sulfonamides J01G: Antibiotics based on cephalosporins J01M: Macrolides, lincosamides, and streptogramins J01XA: Penicillin-based antibiotics J01XE: Other antibiotics J01XXA: Other systemic antibiotics J01XX01: Specific other systemic antibiotics |
MUS: Mid-urethral sling

## Slide 4
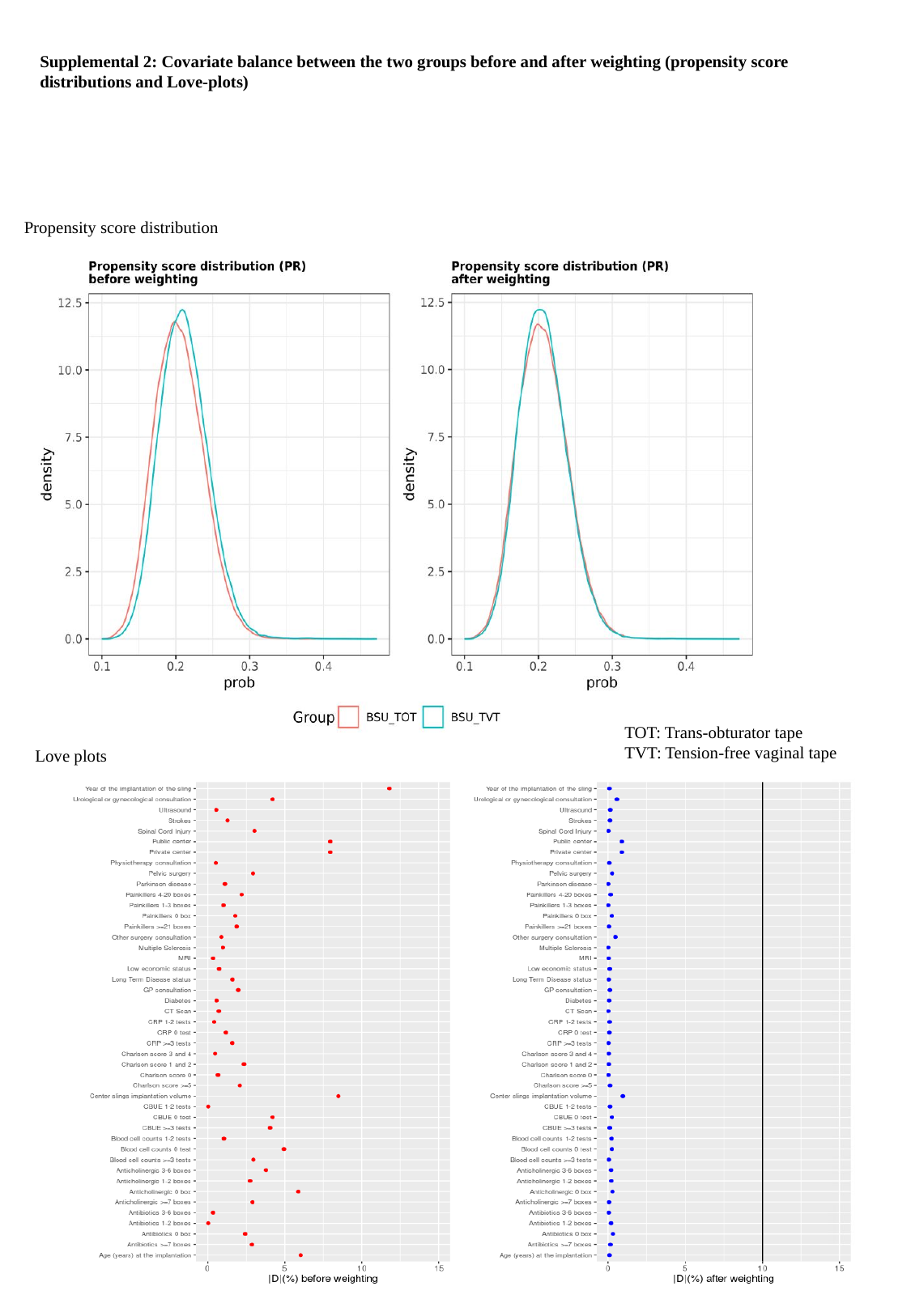

Supplemental 2: Covariate balance between the two groups before and after weighting (propensity score distributions and Love-plots)
Propensity score distribution
TOT: Trans-obturator tape
TVT: Tension-free vaginal tape
Love plots

## Slide 5
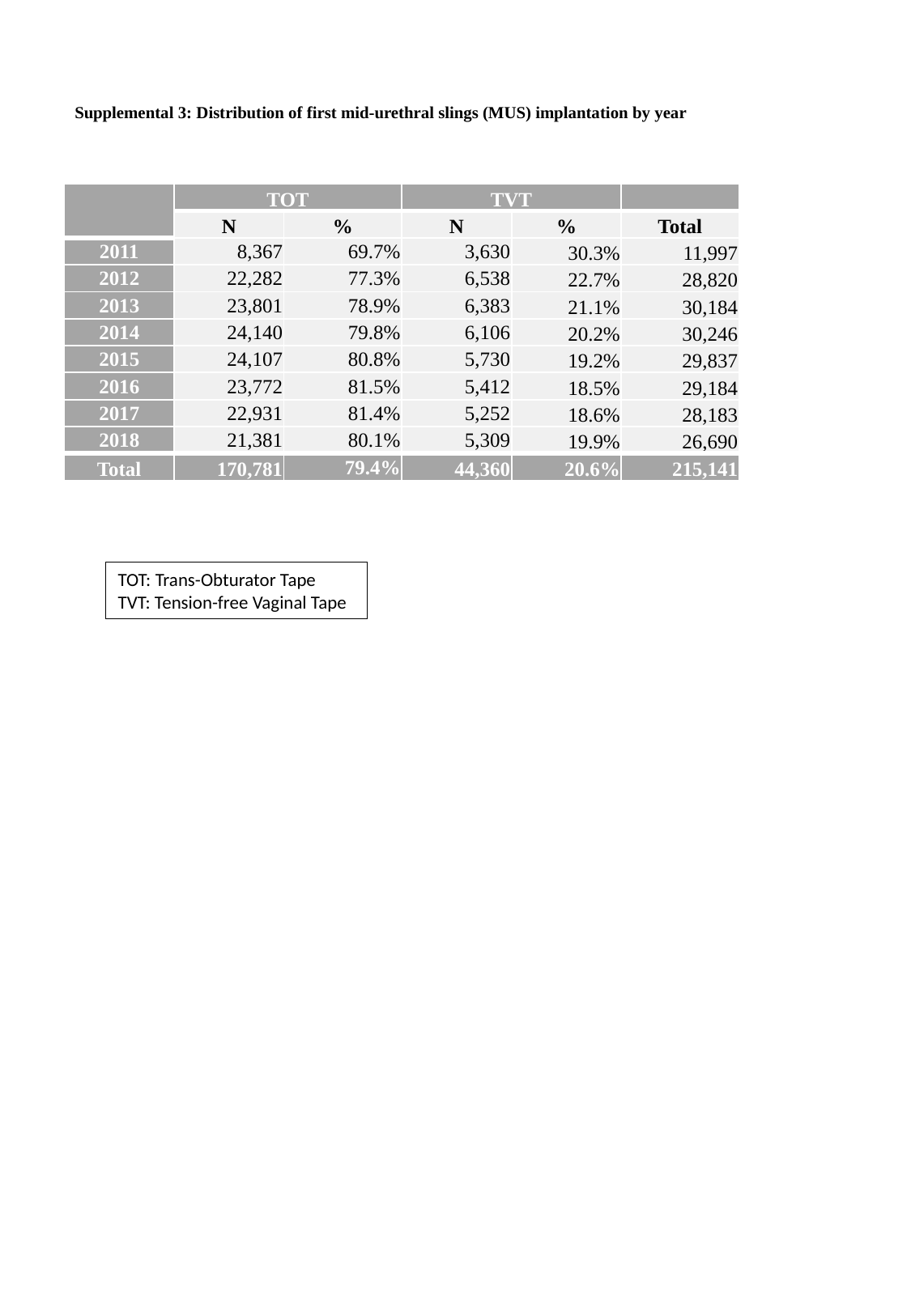

# Supplemental 3: Distribution of first mid-urethral slings (MUS) implantation by year
| | TOT | | TVT | | |
| --- | --- | --- | --- | --- | --- |
| | N | % | N | % | Total |
| 2011 | 8,367 | 69.7% | 3,630 | 30.3% | 11,997 |
| 2012 | 22,282 | 77.3% | 6,538 | 22.7% | 28,820 |
| 2013 | 23,801 | 78.9% | 6,383 | 21.1% | 30,184 |
| 2014 | 24,140 | 79.8% | 6,106 | 20.2% | 30,246 |
| 2015 | 24,107 | 80.8% | 5,730 | 19.2% | 29,837 |
| 2016 | 23,772 | 81.5% | 5,412 | 18.5% | 29,184 |
| 2017 | 22,931 | 81.4% | 5,252 | 18.6% | 28,183 |
| 2018 | 21,381 | 80.1% | 5,309 | 19.9% | 26,690 |
| Total | 170,781 | 79.4% | 44,360 | 20.6% | 215,141 |
TOT: Trans-Obturator Tape
TVT: Tension-free Vaginal Tape

## Slide 6
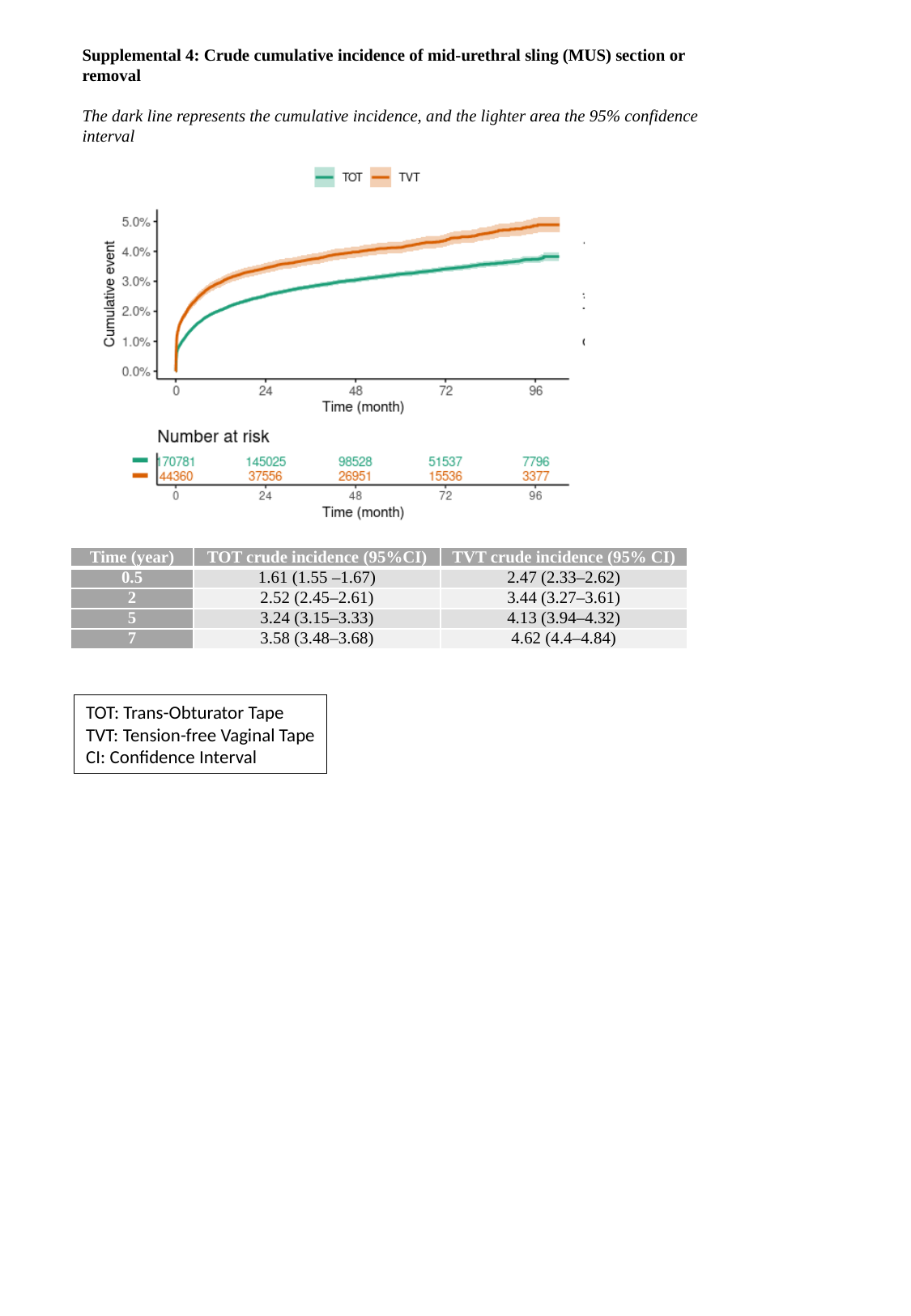

Supplemental 4: Crude cumulative incidence of mid-urethral sling (MUS) section or removal
The dark line represents the cumulative incidence, and the lighter area the 95% confidence interval
| Time (year) | TOT crude incidence (95%CI) | TVT crude incidence (95% CI) |
| --- | --- | --- |
| 0.5 | 1.61 (1.55 –1.67) | 2.47 (2.33–2.62) |
| 2 | 2.52 (2.45–2.61) | 3.44 (3.27–3.61) |
| 5 | 3.24 (3.15–3.33) | 4.13 (3.94–4.32) |
| 7 | 3.58 (3.48–3.68) | 4.62 (4.4–4.84) |
TOT: Trans-Obturator Tape
TVT: Tension-free Vaginal Tape
CI: Confidence Interval

## Slide 7
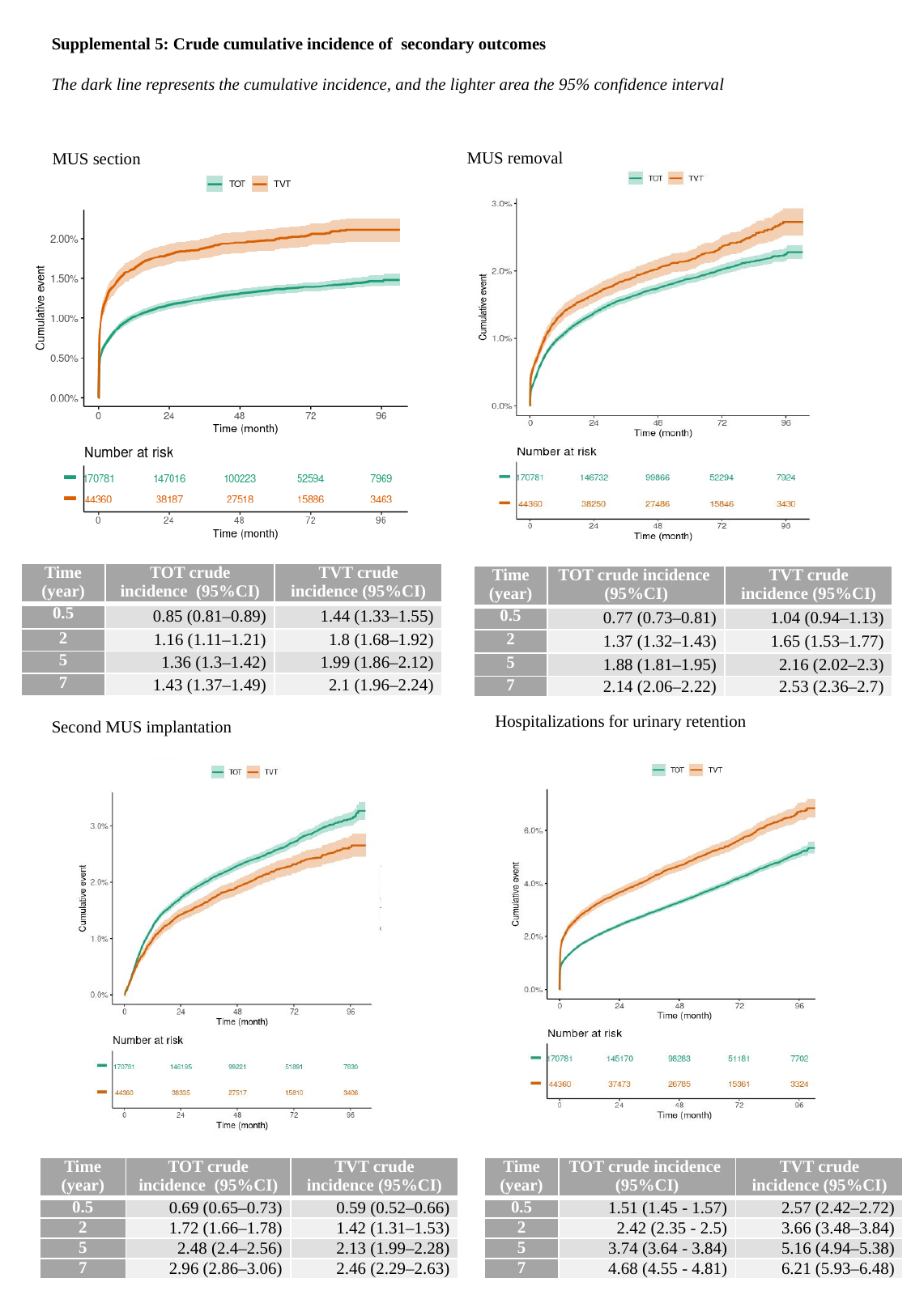

Supplemental 5: Crude cumulative incidence of secondary outcomes
The dark line represents the cumulative incidence, and the lighter area the 95% confidence interval
MUS removal
MUS section
| Time (year) | TOT crude incidence (95%CI) | TVT crude incidence (95%CI) |
| --- | --- | --- |
| 0.5 | 0.85 (0.81–0.89) | 1.44 (1.33–1.55) |
| 2 | 1.16 (1.11–1.21) | 1.8 (1.68–1.92) |
| 5 | 1.36 (1.3–1.42) | 1.99 (1.86–2.12) |
| 7 | 1.43 (1.37–1.49) | 2.1 (1.96–2.24) |
| Time (year) | TOT crude incidence (95%CI) | TVT crude incidence (95%CI) |
| --- | --- | --- |
| 0.5 | 0.77 (0.73–0.81) | 1.04 (0.94–1.13) |
| 2 | 1.37 (1.32–1.43) | 1.65 (1.53–1.77) |
| 5 | 1.88 (1.81–1.95) | 2.16 (2.02–2.3) |
| 7 | 2.14 (2.06–2.22) | 2.53 (2.36–2.7) |
Hospitalizations for urinary retention
Second MUS implantation
| Time (year) | TOT crude incidence (95%CI) | TVT crude incidence (95%CI) |
| --- | --- | --- |
| 0.5 | 0.69 (0.65–0.73) | 0.59 (0.52–0.66) |
| 2 | 1.72 (1.66–1.78) | 1.42 (1.31–1.53) |
| 5 | 2.48 (2.4–2.56) | 2.13 (1.99–2.28) |
| 7 | 2.96 (2.86–3.06) | 2.46 (2.29–2.63) |
| Time (year) | TOT crude incidence (95%CI) | TVT crude incidence (95%CI) |
| --- | --- | --- |
| 0.5 | 1.51 (1.45 - 1.57) | 2.57 (2.42–2.72) |
| 2 | 2.42 (2.35 - 2.5) | 3.66 (3.48–3.84) |
| 5 | 3.74 (3.64 - 3.84) | 5.16 (4.94–5.38) |
| 7 | 4.68 (4.55 - 4.81) | 6.21 (5.93–6.48) |

## Slide 8
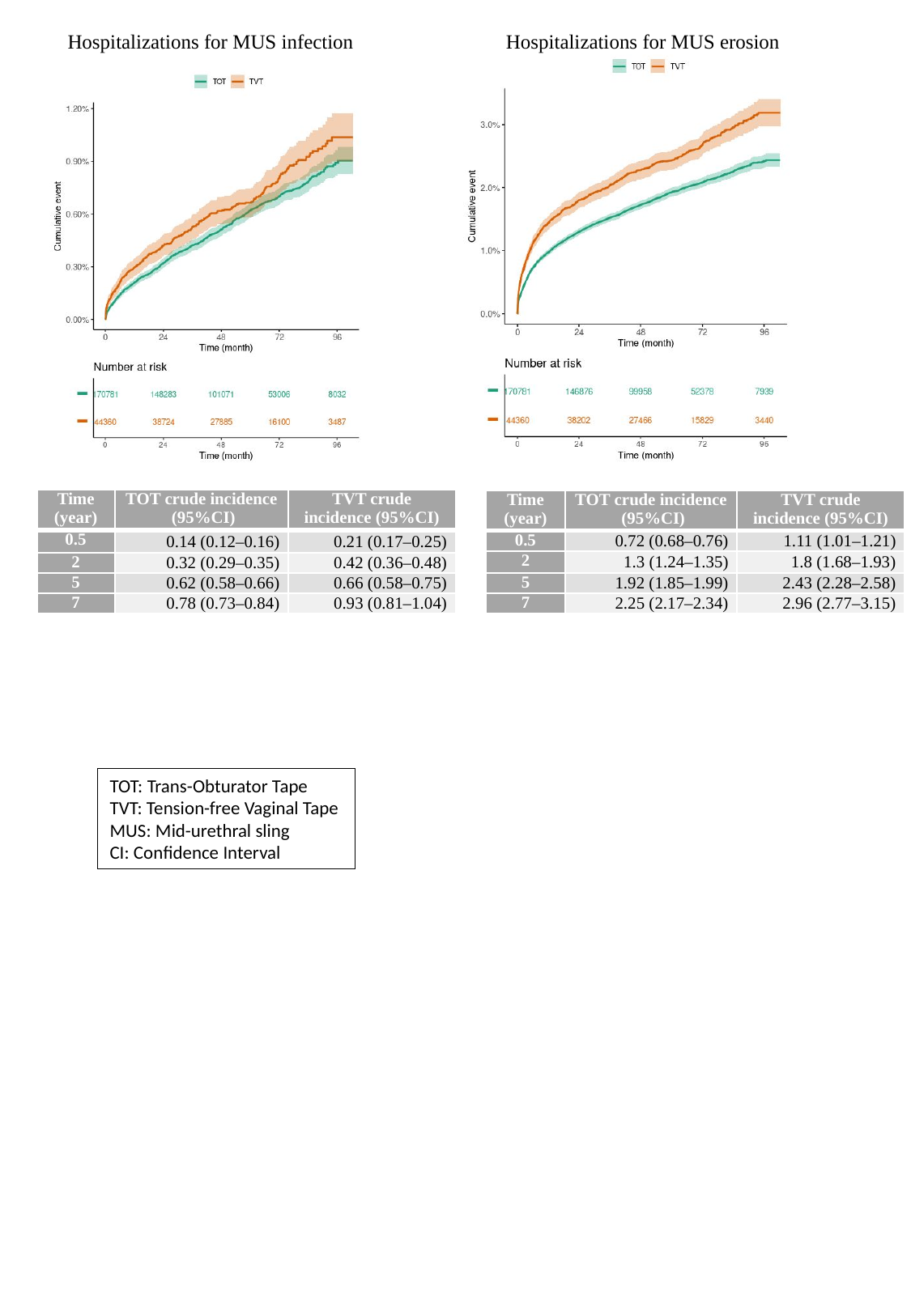

Hospitalizations for MUS erosion
Hospitalizations for MUS infection
| Time (year) | TOT crude incidence (95%CI) | TVT crude incidence (95%CI) |
| --- | --- | --- |
| 0.5 | 0.14 (0.12–0.16) | 0.21 (0.17–0.25) |
| 2 | 0.32 (0.29–0.35) | 0.42 (0.36–0.48) |
| 5 | 0.62 (0.58–0.66) | 0.66 (0.58–0.75) |
| 7 | 0.78 (0.73–0.84) | 0.93 (0.81–1.04) |
| Time (year) | TOT crude incidence (95%CI) | TVT crude incidence (95%CI) |
| --- | --- | --- |
| 0.5 | 0.72 (0.68–0.76) | 1.11 (1.01–1.21) |
| 2 | 1.3 (1.24–1.35) | 1.8 (1.68–1.93) |
| 5 | 1.92 (1.85–1.99) | 2.43 (2.28–2.58) |
| 7 | 2.25 (2.17–2.34) | 2.96 (2.77–3.15) |
TOT: Trans-Obturator Tape
TVT: Tension-free Vaginal Tape
MUS: Mid-urethral sling
CI: Confidence Interval

## Slide 9
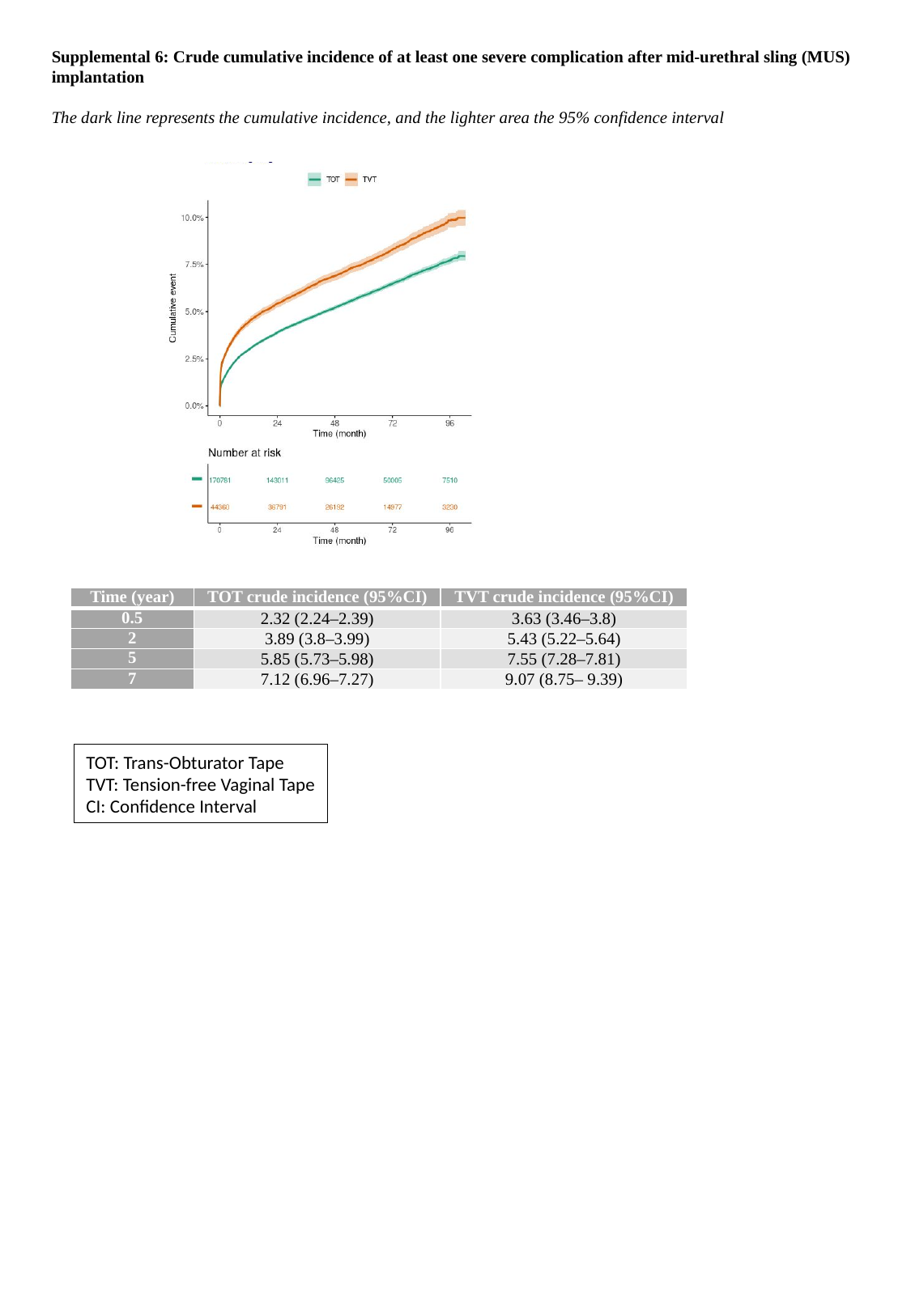

Supplemental 6: Crude cumulative incidence of at least one severe complication after mid-urethral sling (MUS) implantation
The dark line represents the cumulative incidence, and the lighter area the 95% confidence interval
| Time (year) | TOT crude incidence (95%CI) | TVT crude incidence (95%CI) |
| --- | --- | --- |
| 0.5 | 2.32 (2.24–2.39) | 3.63 (3.46–3.8) |
| 2 | 3.89 (3.8–3.99) | 5.43 (5.22–5.64) |
| 5 | 5.85 (5.73–5.98) | 7.55 (7.28–7.81) |
| 7 | 7.12 (6.96–7.27) | 9.07 (8.75– 9.39) |
TOT: Trans-Obturator Tape
TVT: Tension-free Vaginal Tape
CI: Confidence Interval

## Slide 10
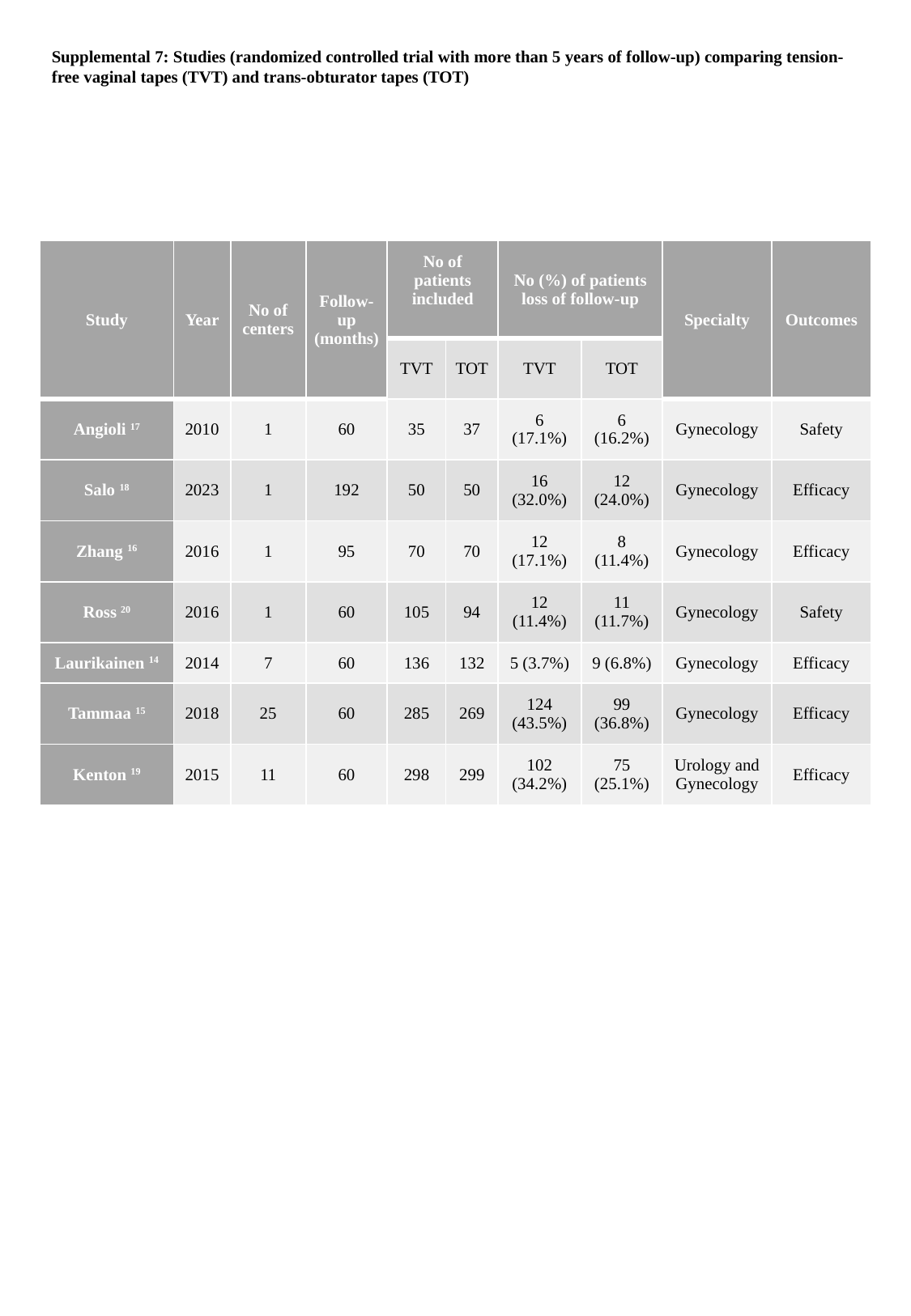

Supplemental 7: Studies (randomized controlled trial with more than 5 years of follow-up) comparing tension-free vaginal tapes (TVT) and trans-obturator tapes (TOT)
| Study | Year | No of centers | Follow-up (months) | No of patients included | | No (%) of patients loss of follow-up | | Specialty | Outcomes |
| --- | --- | --- | --- | --- | --- | --- | --- | --- | --- |
| | | | | TVT | TOT | TVT | TOT | | |
| Angioli 17 | 2010 | 1 | 60 | 35 | 37 | 6 (17.1%) | 6 (16.2%) | Gynecology | Safety |
| Salo 18 | 2023 | 1 | 192 | 50 | 50 | 16 (32.0%) | 12 (24.0%) | Gynecology | Efficacy |
| Zhang 16 | 2016 | 1 | 95 | 70 | 70 | 12 (17.1%) | 8 (11.4%) | Gynecology | Efficacy |
| Ross 20 | 2016 | 1 | 60 | 105 | 94 | 12 (11.4%) | 11 (11.7%) | Gynecology | Safety |
| Laurikainen 14 | 2014 | 7 | 60 | 136 | 132 | 5 (3.7%) | 9 (6.8%) | Gynecology | Efficacy |
| Tammaa 15 | 2018 | 25 | 60 | 285 | 269 | 124 (43.5%) | 99 (36.8%) | Gynecology | Efficacy |
| Kenton 19 | 2015 | 11 | 60 | 298 | 299 | 102 (34.2%) | 75 (25.1%) | Urology and Gynecology | Efficacy |
